# Advent of severe mitral regurgitation in surgical ventricular restoration and ventricular septal rupture repair

**DOI:** 10.1186/s12872-023-03537-9

**Published:** 2023-10-12

**Authors:** Yihua Liu, Laura Filippetti, Pan Dan, Elodie Phamisith, Valério Sanesi, Damien Mandry, Giuseppe Lauria, Juan Pablo Maureira

**Affiliations:** 1 Department of Cardiovascular Surgery and heart transplantation, University Hospital of Nancy, University of Lorraine, Vandoeuvre-les-Nancy, 54500 France; 2 Department of Cardiology, University Hospital of Nancy, University of Lorraine, Vandoeuvre-les-Nancy, 54500 France; 3 Department of Radiology, University Hospital of Nancy, University of Lorraine, Vandoeuvre-les-Nancy, 54500 France

**Keywords:** Mitral regurgitation, Postinfarction ventricular septal rupture, Surgical ventricular restoration

## Abstract

**Background:**

Per-procedural severe mitral regurgitation is a rare complication in concomitant surgical ventricular restoration and postinfarction ventricular septal rupture repair. It is challenging to discover the underlying etiology and adopt an appropriate strategy, in particular, in a high-risk patient.

**Case presentation:**

Semi-emergent surgical ventricular restoration combined with ventricular septal rupture closure and coronary artery bypassing was performed in a 67-year-old male patient. Severe mitral regurgitation was detected after the weaning of cardiopulmonary bypass. Two key questions arose in the management of this condition: did the regurgitation exist previously and was dissimulated by significant left-to-right shunt, or it occurred secondarily to the Dor procedure? Which was the better management strategy, chordal-sparing mitral valve replacement or mitral plasty? We believed that severe mitral regurgitation was under-estimated pre-operatively and we performed an downsizing annuloplasty to treat mitral regurgitation. The outcomes were promising and the patient did well in follow-up.

**Conclusions:**

Our case brought out an open discussion on the etiology and therapeutic strategies of this complicated condition.

## Background

Functional mitral regurgitation (MR) might co-exist with but be dissimulated by post-infarction ventricular septal rupture (VSR), especially in case of a significant left-to-right shunt; the severity of MR could not be precisely evaluated in pre-operative work-up until surgical repair of the VSR. It remains controversial whether downsizing annuloplasty or mitral valve replacement is the better strategy in the management of ischemic MR.

## Case presentation

**Fig. 1:**
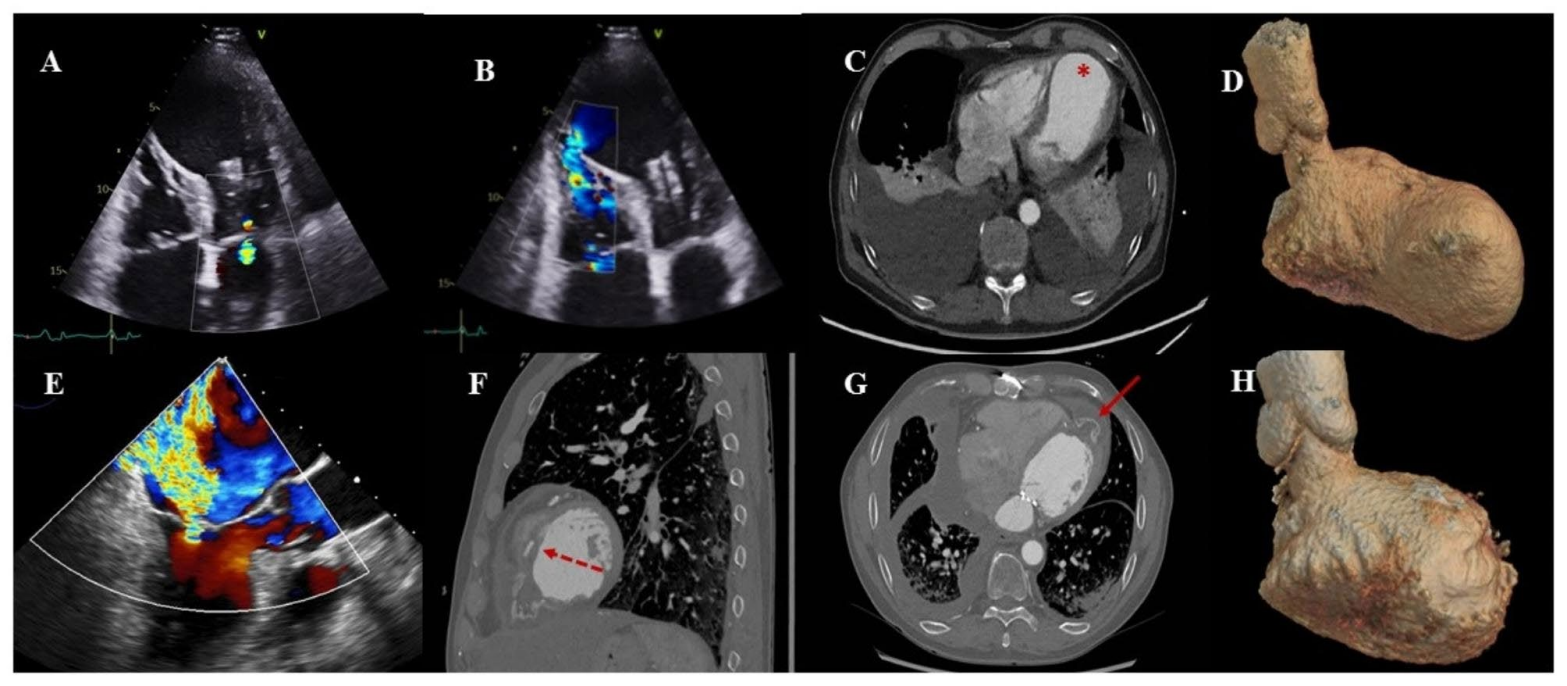
Preoperative transthoracic echocardiography visualized a mild mitral regurgitation (**A**) and a ventricular septal rupture (**B**); preoperative computerized Tomography Angiography (CTA) showed a large left ventricular aneurysm (*, **C, D**); intraoperative transesophageal echocardiography uncovered a severe mitral regurgitation at the first attempt of weaning of cardiopulmonary bypass (**E**); CTA in 6 months showed a successful repair of ventricular septal rupture with bovine pericardial patch (arrow with dotted line, **F**), an exclusion of left ventricular aneurysm with Teflon felt (arrow with solid line, **G, H**) and an almost normal LV morphology (**H**)

A 67-year-old male patient consulted the emergency department for intermittent back pain and aggravation of exertional dyspnea since 4 months. Physical examination revealed an arterial pressure of 106/82 mmHg, heart rates of 94 beats per minute, and a bilateral lower limb edema. He weighted 79 kg and his height was 181 cm. A rumbling 3/6 heart murmur was heard at the 4th left intercostal space. The biological exams showed a five-fold elevation of high sensitive Troponin-I and a 700-fold elevation of NT-pro brain natriuretic peptide, a moderate renal insufficiency with creatinine clearance rates of 55ml/min.m², and signs of tissue hypoperfusion with lactate level of 2.1mmol/L. The electrocardiogram showed a sinus rhythm and an ST-elevation in the leads V2-V3. The transthoracic echocardiography showed a dilated left ventricle (left ventricular end diastolic diameter of 68 mm) and severely depressed ejection fraction of 29% with a large antero-apical aneurysm which was confirmed by Computerized Tomography Angiography (Fig. 1C and D); a ventricular septal defect measured 8 mm was also visualized (Fig. 1B). Cardiac index was calculated to be 1.4 L/min/m². Mitral regurgitation was mild (grade I, Fig. 1A), and right ventricular dysfunction was severe with tricuspid annular plane systolic excursion (TAPSE) of 7 mm. Systolic pulmonary artery pressure was estimated to be 63mmHg. Cardiac magnetic resonance imaging (MRI) confirmed the ventricular septal defect with an estimated ratio pulmonary/systemic flow of 3.6. The extent of transmural infarction in the left ventricle was assessed to be 55%. Coronary angiography uncovered a sub-acute occlusion from the middle of the left anterior descending artery, a chronic occlusion of the ostium of the 3rd obtuse marginal artery as well as a significant stenosis of the right coronary artery. The EuroSCORE II was 30.8%.

After panel discussion and medical preparation including Levosimendan infusion, a semi-urgent operation was scheduled which consisted of VSR closure, surgical ventricular restoration and right coronary artery bypass grafting. After harvesting of the great saphenous vein, full sternotmy and dividing the dense inter-pericardial adhesions, the heart was put onto cardiopulmonary bypass (CPB) with cannulation of the ascending aorta, and superior and inferior vena cava. The distal ascending aorta was then cross clamped, the right coronary artery bypass grafting was performed using great saphenous vein; left ventriculotomy was thereafter made through apical aneurysm, the demarcation between healthy myocardium and scarred tissue was obvious and there was no involvement of the mitral subvalvular apparatus; a muscular VSD sized 8 mm*10mm was then identified and repaired using bovine pericardial patch; finally, ventricular restoration was accomplished according to the Dor procedure. The first weaning cardiopulmonary bypass was uneventful; however, intraoperative transesophageal echocardiography (TOE) uncovered a severe type IIIb mitral regurgitation (MR, Fig. 1E) which did not exist preoperatively. A decision of re-clamping was soon made to perform mitral valve plasty using a downsized ring (Edwards Physio II, 28 mm). The subsequent TOE of control showed a trial residual MR with a mean transvalvular pressure gradient of 3mmHg. The second CPB was weaned with multiple inotropic supports but without mechanical circulatory assist. The total aortic cross clamping time and CPB time were 190 and 270 min, respectively.

The postoperative course was complicated with, in particular, an hypoxemia necessitating a delayed extubation (Day 1) and high-flow oxygen therapy, an acute renal failure requiring continuous renal replacement therapy during 4 days, a sepsis complicating a bacteremia treated with antibiotics over 2 weeks, and a bilateral pleural effusion managed with pleurocentesis twice. Nevertheless, the patient did not present with severe low cardiac output syndrome and the catecholamines were progressively weaned on Day 11. After 30-day stay in intensive care unit and 6 days in the ward, the patient was finally discharged for rehabilitative training. At discharge, transthoracic echocardiography of control showed left ventricular dimension of normal range with an ejection fraction of 40% and cardiac index of 2.4 L/min.m², a good result of mitral plasty with a mean transvalvular pressure gradient of 2mmHg. The TAPSE was assessed to be 12 mm and systolic pulmonary pressure 23 mmHg. During eighteen months following this major operation, he was doing well, the computerized tomography angiography in follow up showed an almost-normal left ventricular morphology (Fig. 1F and G H), and transthoracic echocardiography of control showed no signs of MR recurrence and a preserved left ventricular ejection of 50%.

## Discussion

This case represents a rare and challenging condition, which requires simultaneous surgical management of three major mechanical complications following myocardial infarction; namely, ventricular septal rupture, left ventricular aneurysm and ischemic mitral regurgitation. Besides the technical and strategic merits, this case brings a discussion on the etiology of severe MR appearing at the weaning of the first CPB. One probable and more likely explanation is that this MR existed already, but was dissimulated by a significant left-to-right shunt (Qp/Qs = 3.6) in preoperative evaluation; severe MR was uncovered once the ventricular septal rupture was repaired. Another less likely hypothesis is that the MR was aggravated by surgical left ventricular remodeling (the Dor procedure), which might result in LV distortion and papillary muscle displacement. However, according to perprocedural exploration, the scar tissue and the stitches were away from mitral subvalvular apparatus; in addition, the mechanism of MR was the restriction of mitral leaflets, which was repaired with downsizing annuloplasty.

Despite the advancements in medical and percutaneous management for postinfarction VSR, the mortality remains high even in patients undergoing surgical repair [1]. The timing of surgery is an important issue, which is determined by the fragility of myocardial scar and end organs’ tolerance to low cardiac output status. Ventricular contractile reserve and its impact on haemodynamics are important factors for decision-making and prognosis prediction. This case raises a concern with regard to appropriate preoperative evaluation of LV contractile function in case of complex anatomical anomalies. Generally, the volume-derived parameters such as ejection fraction and cardiac index underestimate ventricular contractile reserve in a large LV aneurysm but overestimate it in a left-to-right shunt. In our case, the patient experienced an uneventful weaning of CPB despite of long-lasting aortic cross clamping and a remarkable improvement of ejection fraction (40% vs. 29%) shortly after surgery. A posteriori, the preoperative LV systolic function was underestimated using volume-derived parameters. Should strain and strain rate imaging by echocardiography be a more pertinent method for assessment of myocardial function in this case? Strain imaging allows quantifying regional wall contractility and global myocardial dyssynchrony, thus provides valuable prognostic information, especially in patients undergoing myocardial revascularization and left ventricular restoration surgery [2].

It’s worth noting that the decision-making for per-procedural severe mitral regurgitation was indeed a dilemma between mitral plasty with a downsized annuloplasty ring and valve replacement with chordal sparing. A growing body of evidence favors chordal-sparing mitral valve replacement over annuloplasty in surgical management of ischemic mitral regurgitation, since the former trends to be associated with better midterm outcomes due to in part a lower incidence of moderate-to-severe mitral regurgitation in the processes of LV reverse remodeling after surgery [3]. Whereas, the scenario in our case was particular, including “new-onset” severe MR, concomitant ventricular restoration surgery and feasibility of downsized annuloplasty. We believed that mitral plasty could provide a better hemodynamics and satisfactory outcomes in this condition. The early results and ongoing follow-up are promising.

In conclusion, this rare case shows that, as a severe mechanical complication associated with myocardial infarction, severe mitral regurgitation might be dissimulated by important left-to-right shunt, or occur secondarily to papillary muscle displacement following surgical ventricular restoration. Volume-derived parameters fail to quantify precisely ventricular contractile reserve in complex anatomical anomalies. Downsized annuloplasty remains a promising surgical strategy in the management of ischemic mitral regurgitation.

## Data Availability

Data sharing is not applicable to this article as no datasets were generated.

## Abbreviations

MR: Mitral regurgitation
VSR: Ventricular septal rupture
TAPSE: Tricuspid annular plane systolic excursion
MRI: Magnetic resonance imaging
CPB: Cardiopulmonary bypass
VSD: Ventricular septal defect
TEE: Transesophageal echocardiography
LV: Left ventricle
